# BAPoma, ein seltener Naevus als Schlüssel zur Diagnose des *BAP1*‐assoziierten Tumorprädisposition‐Syndroms

**DOI:** 10.1111/ddg.15791_g

**Published:** 2025-10-23

**Authors:** Lara Racz, Sandra Pasternack‐Ziach, Isabel Spier, Stephan Forchhammer, Claudia Rehkämper, Peter Kind, Julia Reifenberger, Silke Redler

**Affiliations:** ^1^ Institut für Humangenetik Medizinische Fakultät und Universitätsklinikum Düsseldorf; ^2^ Institut für Humangenetik Universität Bonn Medizinische Fakultät und Universitätsklinikum Bonn; ^3^ Nationales Zentrum für erbliche Tumorsyndrome Universitätsklinikum Bonn; ^4^ Klinik für Dermatologie Universität Tübingen; ^5^ Dermatopathologie Offenbach am Main; ^6^ Klinik für Dermatologie Medizinische Fakultät und Universitätsklinikum Düsseldorf Heinrich‐Heine‐Universität Düsseldorf

Sehr geehrte Herausgeber,

Eine 23‐jährige Frau stellte sich aufgrund größenprogredienter Nävi in unserem Klinikum vor. Es wurde ein Hautkrebsscreening mit *body mapping* durchgeführt. Aus dem Bereich der dorsalen beziehungsweise ventralen Flanke wurden drei suspekte Nävi exzidiert. Bei zwei dieser drei suspekten Hautveränderungen handelte es sich um junktionale Nävi. Die histologische Untersuchung des Gewebes aus der ventralen Flanke ergab jedoch einen melanozytären Naevus mit partieller *BAP1*‐Inaktivierung (BAPoma) (Abbildung 1). Immunhistochemisch zeigte sich ein Verlust der nukleären *BAP1*‐Expression (Abbildung 1). Aus peripheren Blutleukozyten wurde DNS extrahiert und in Einklang mit dem deutschen Gendiagnostikgesetz eine Sequenzierung sowie ein Deletions‐/Duplikationsscreening des Gens BRCA1‐assoziiertes Protein 1 (*BAP1*) durchgeführt. Dabei wurde die heterozygote pathogene Keimbahnvariante c.1813G>T;p.(Glu605Ter) festgestellt, die zu einem vorzeitigen Stoppcodon führt. Der Nachweis dieser Variante sicherte die Diagnose eines *BAP1*‐assoziierten Tumorprädisposition‐Syndroms (BAP1‐TPS) bei der jungen Frau. Bei Aufnahme des Familienstammbaums über drei Generationen ergab sich eine Häufung von Tumorerkrankungen in der väterlichen Familie der Index‐Patientin, die sich teilweise in einem frühen Erwachsenenalter manifestiert hatten (Abbildung 2). Interessanterweise wurden bei der Cousine väterlicherseits (III:3) im Alter von 23 Jahren zwei suspekte Hautläsionen exzidiert, die sich beide als BAPome herausstellten. Beim Vater (II:1) wurde im Alter von 47 Jahren die Diagnose eines Urothelkarzinom der Harnblase gestellt, in der Folge trat ein Rezidiv auf. Im Alter von 54 Jahren wurde ein Pleuramesotheliom diagnostiziert. Darüber hinaus hatte sich der Vater der Entfernung zahlreicher Basalzellkarzinome unterzogen. Bei ihm wurde die bei seiner Tochter identifizierte heterozygote pathogene Keimbahnvariante c.1813G>T;p.(Glu605Ter) im *BAP1*‐Gen ebenfalls festgestellt und damit die Diagnose eines BAP1‐TPS bei ihm gestellt. Bei der Tante väterlicherseits (II:2) wurden seit ihrem 47. Lebensjahr 50 bis 60 adenomatöse Kolonpolypen entfernt, eine pathogene Keimbahnvariante in bekannten Polyposis‐assoziierten Suszeptibilitätsgenen konnte bei ihr nicht nachgewiesen werden. Zwischen dem 40. und 45. Lebensjahr wurden bei ihr mindestens drei Basalzellkarzinome im Halsbereich entfernt. Der Großvater väterlicherseits ist im Alter von 75 Jahren an einem Nierenzellkarzinom gestorben, dessen Schwester verstarb im Alter von 41 Jahren an metastasierendem Brustkrebs. Eine Segregation mit der familiären *BAP1*‐Variante war bei den verstorbenen Angehörigen nicht mehr möglich. Mit Ausnahme eines Cousins väterlicherseits (III:4) trugen jedoch alle sonst getesteten Familienmitglieder die familiäre *BAP1*‐Variante (Abbildung 2).

**ABBILDUNG 1:**
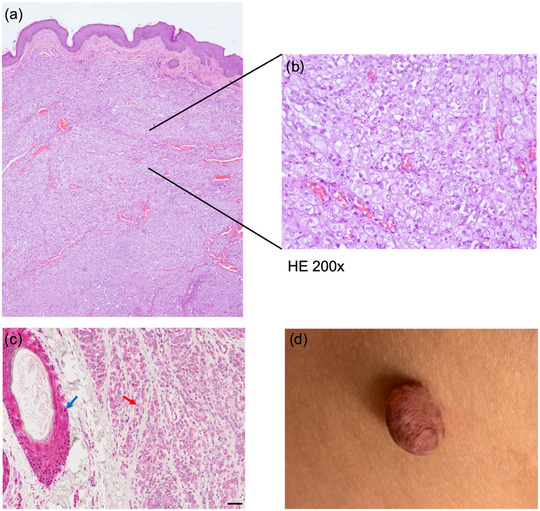
Histologie, immunhistochemische Färbung für das BRCA1‐assoziiertes Protein‐1 (BAP1), klinisches Bild des BAPoms. (a) Unter einer unauffälligen Epidermis befindet sich im Korium zahlreiches Tumorgewebe mit nodulärem Wachstumsmuster, das von zahlreichen Blutgefäßen durchzogen ist. (b) Bei stärkerer Vergrößerung sind Nester und Stränge relativ monomorpher epitheloider Zellen mit isolierten Mitosen zu erkennen (oben rechts). Diese Histologie ist mit einer partiellen BAP1‐Inaktivierung vereinbar (Hämatoxylin‐Eosin‐Färbung, Originalvergrößerung x 200). (c) Naevuskomponenten zeigen einen Verlust der Kernexpression für BAP1 (roter Pfeil). Das interne Kontrollgewebe zeigt die erhaltene Kernexpression im Bereich des Haarfollikelepithels (blauer Pfeil) (Maßstabsbalken: 50 µm). (d) Circa 0,7 cm großer, gestielter, unregelmäßig bräunlich pigmentierter Tumor an der ventralen Flanke.

**ABBILDUNG 2:**
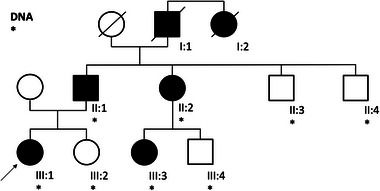
Familienstammbaum. Personen, die von bösartigen oder gutartigen Tumoren betroffen sind, werden mit schwarzen Kreisen (weiblich) und Quadraten (männlich) dargestellt. Ein Strich durch das Symbol zeigt an, dass die betreffende Person verstorben ist. Sternchen kennzeichnen Personen, für die DNA verfügbar war. Die Indexpatientin (III:1) ist mit einem Pfeil gekennzeichnet. Mit Ausnahme eines Cousins väterlicherseits (III:4) trugen alle getesteten Personen die *BAP1*‐Variante c.1813G>T;p.(Glu605Ter). Großvater väterlicherseits (I:1): Nierenzellkarzinom, gestorben im Alter von 75 Jahren; Schwester des Großvaters väterlicherseits (I:2): metastasierendes Mammakarzinom, gestorben im Alter von 41 Jahren; Vater (II:1): Urothelkarzinom der Harnblase, diagnostiziert im Alter von 47 Jahren, 4 Jahre später gefolgt von einem metastasierenden Rezidiv. Pleuramesotheliom, diagnostiziert im Alter von 54 Jahren. Entfernung zahlreicher Basalzellkarzinome; Tante väterlicherseits (II:2): Exzision von 50 bis 60 adenomatösen Kolonpolypen seit dem Alter von 47 Jahren, Entfernung von mindestens drei Basalzellkarzinomen im Halsbereich im Alter von 40 bis 45 Jahren; Onkel väterlicherseits (II:3) im Alter von 56 Jahren nicht erkrankt; Onkel väterlicherseits (II:4) im Alter von 58 Jahren nicht erkrankt; Index (III:1) melanozytärer Naevus mit partieller BAP1‐Inaktivierung (BAPoma) an der rechten Flanke im Alter von 22 Jahren; Schwester (III:2) im Alter von 19 Jahren nicht betroffen; Cousine väterlicherseits (III:3) zwei BAPomas (Bauch, Hals) im Alter von 23 Jahren; Cousin väterlicherseits (III:4) im Alter von 28 Jahren nicht betroffen.

Das BAP1‐assoziierte Tumorprädisposition‐Syndrom wurde erstmalig 2011 beschrieben.^1^ Dieses seltene familiäre Krebssyndrom wird autosomal‐dominant vererbt und beruht auf pathogenen *BAP1*‐Keimbahnvarianten, dessen Hauptfunktion die Tumorsuppression auf molekularer Ebene ist. *BAP1* wurde aufgrund seiner Interaktion mit dem Gen *BRCA1* (BReast CAncer Gene 1) benannt, das durch die Reparatur von DNA‐Doppelstrangbrüchen genomische Stabilität gewährleistet.^2^ *BAP1* wird an Stellen von Doppelstrangbrüchen rekrutiert und unterstützt deren Reparatur durch homologe Rekombination, indem es die Anlagerung von BRCA1 erleichtert.^3^ Darüber hinaus ist es Bestandteil des Polycomb‐Repressoren‐Deubiquitinase‐Komplexes, der Ubiquitin von Histonen (H2AK119ub) entfernt und dadurch die Transkription reguliert. Ein Verlust von BAP1 stört diesen Prozess, was zu einer veränderten Expression von Genen führt, die in der Zellzykluskontrolle, DNA‐Reparatur und im Zellmetabolismus eine Rolle spielen.^4^ BAP1‐TPS prädisponiert für verschiedene Tumorentitäten, gutartige melanozytäre Hauttumoren sowie zahlreiche bösartige Tumoren in verschiedenen Organsystemen.^5^ Wie bei vielen seltenen TPS ist unklar, welche Krebsarten eindeutig mit dem BAP1‐TPS assoziiert sind. Es besteht Konsens, dass der Kernphänotyp des BAP1‐TPS das BAPom, das kutane Melanom, das Aderhautmelanom, das Basalzellkarzinom, das maligne Mesotheliom der Pleura und des Peritoneums sowie das Nierenzellkarzinom umfasst. Darüber hinaus scheint es möglich, dass zu dem Spektrum von BAP1‐TPS auch Meningeome, Cholangiokarzinome, Brust‐, Harnblasen‐ und Lungenkrebs zählen.^1^, ^6^, ^7^ Das BAPom ist häufig die Erstmanifestation dieses seltenen TPS.

Klinisch erscheinen *BAP1*‐inaktivierte Nävi als kuppelförmige, hautfarbene oder rötliche Papeln. Histologisch stellen sie sich typischerweise als dermale melanozytäre Proliferationen dar, die mit prominenten epitheloiden Zellen kombiniert sind. Letztere können einen nekrotischen Pleomorphismus und reichlich amphophiles Zytoplasma aufweisen und zeigen immunhistochemisch einen Verlust der nukleären BAP1‐Expression.^8^, ^9^ Dermatologen sind ideal positioniert, um spezifische klinische Merkmale zu erkennen und eine Keimbahntestung und genetische Beratung zu veranlassen. Dies ist von entscheidender Bedeutung für die Identifizierung von Hochrisikopersonen und deren Einschluss in Screening‐Programme, die darauf abzielen, bösartige Tumoren in einem frühen Entwicklungsstadium zu erkennen und Krebserkrankungen „abzufangen“, bevor sie aggressiv werden. Im Jahr 2023 veröffentlichte ein europäisches Expertengremium detaillierte Empfehlungen zum Umgang mit dem BAP1‐TPS, in denen die Notwendigkeit regelmäßiger Haut‐ und Augenscreenings sowie Bildgebungen der Nieren hervorgehoben wurde.^10^ Das im vorliegenden Bericht beschriebene Tumorspektrum erweitert die phänotypische Variabilität von BAP1‐TPS. Insbesondere ist dies der erste BAP1‐TPS‐Bericht, in dem kolorektale adenomatöse Polypen (II:2) beschrieben werden, obwohl wir die Möglichkeit nicht ausschließen können, dass sich die kolorektalen Polypen unabhängig von der familiären BAP1‐Variante entwickelt haben.

Der vorliegende Fall zeigt die Schwierigkeiten, die mit der Charakterisierung seltener Tumorsyndrome verbunden sind, für die nur begrenzte wissenschaftliche Erkenntnisse vorliegen. Die Charakterisierung des assoziierten Tumorspektrums, die Ermittlung entsprechender Genotyp‐Phänotyp‐Korrelationen und die Erforschung des Nutzens von Überwachung und klinischem Management sind entscheidende Schritte nach vorn. Zusammenfassend lässt sich sagen, dass unser Bericht eine BAP1‐TPS‐Familie mit kutanen und nichtkutanen Tumormanifestationen beschreibt. BAP1‐TPS ist selten, und aufgrund seines vielfältigen Phänotyps – auch innerhalb von Familien – kann die Diagnose eines BAPoms ein wertvolles Instrument für die Identifizierung dieses Tumorsyndroms darstellen. Die frühzeitige Erkennung von BAP1‐TPS erleichtert die Einleitung von Früherkennungsmaßnahmen, um Malignome zu verhindern, die bei Diagnosestellung weit fortgeschritten sind.

## DANKSAGUNG

Wir danken der Familie für ihr Einverständnis, ihren Fall veröffentlichen und histologische/klinische Bilder zeigen zu dürfen.

Open access Veröffentlichung ermöglicht und organisiert durch Projekt DEAL.

## INTERESSENKONFLIKT

Keiner.
